# Association between dietary intake of one-carbon metabolism nutrients and hyperglycemia in coal-burning fluorosis areas of Guizhou, China

**DOI:** 10.3389/fnut.2022.1002044

**Published:** 2022-10-10

**Authors:** Li Ding, Qinglin Yang, Zhongming Sun, Lu Liu, Zeyu Meng, Xun Zhao, Na Tao, Jun Liu

**Affiliations:** ^1^Department of Preventive Medicine, School of Public Health, Zunyi Medical University, Zunyi, China; ^2^Department of Nutrition and Food Hygiene, School of Public Health, Soochow University, Suzhou, China; ^3^Department of Chronic Disease, Center of Disease Control and Prevention of Zhijin County, Bijie, China; ^4^Department of Pharmacy, Affiliated Hospital of Zunyi Medical University, Zunyi, China

**Keywords:** one-carbon metabolism, hyperglycemia, betaine, choline, methionine, folate, vitamin B_6_, vitamin B_12_

## Abstract

**Background and aims:**

There are limited studies describing the association between dietary intake of one-carbon metabolism nutrients and hyperglycemia. The present study aimed to investigate the association of habitual dietary intake of one-carbon metabolism nutrients with hyperglycemia in a fluorosis area in China, and explored the interaction between these nutrients and fluorosis related to hyperglycemia.

**Method:**

In a cross-sectional study, we recruited 901 villagers, ages ranging from 18–75, in Guizhou Province. Dietary data and other covariate data were obtained through an interviewer-administered questionnaire. We collected venous blood samples from participants who had fasted for one night to obtain fasting blood glucose levels and we categorized dietary intake of betaine, total choline, methionine, folate, vitamins B_6_ and B_12_, and choline subclasses into quartiles (Q1–Q4). The lowest quartile (Q1) served as the reference group. An unconditional logistic regression model was used to evaluate the protective effects of a dietary intake of one-carbon nutrients against hyperglycemia. We calculated Odds Ratios (ORs) with 95% confidence intervals (CIs). A presence or absence of fluorosis subgroup analysis was performed to determine the potential effect of fluorosis on hyperglycemia.

**Result:**

After adjusting for potential confounding factors, we found that a greater intake of dietary vitamin B_6_, total choline and methyl-donor index was inversely associated with the occurrence of hyperglycemia (*P*-trend <0.05). However, there were no significant associations between hyperglycemia and the dietary intake of folate, vitamin B_12_, methionine, and betaine. As for the choline subgroups, it showed that the dietary intake of free choline, phosphatidylcholine, and glycerol phosphatidylcholine was negatively correlated with the occurrence of hyperglycemia (*P* < 0.05). In contrast, there was no statistical association between dietary phosphatidylcholine and sphingomyelin and hyperglycemia (all *P* > 0.05). The results of subgroup analysis showed that dietary intake of folate, vitamin B_6_, total choline, free choline, glycerol phosphorylcholine, and phosphocholine had a protective effect against the occurrence of hyperglycemia in the non-fluorosis subgroup, although no effects were observed in the fluorosis subgroup. There were significant interactions between these nutrients and fluorosis (*P* = 0.010–0.048).

**Conclusion:**

The study demonstrated that higher dietary intake of vitamin B_6_, total choline, methyl-donor index, free choline, glycerol phosphorylcholine, and phosphocholine in choline compounds were associated with a lower incidence of hyperglycemia. Moreover, the associations were modified by the presence or absence of fluorosis. Further investigation is needed to test the association in large-scale follow-up studies.

## Introduction

Hyperglycemia is an asymptomatic disease involving the development of cardiovascular problems and diabetes. According to the 2017 Global Burden of Disease study, about 1.02 million died from diabetes worldwide; 15.09 million died of cardiovascular and cerebrovascular diseases, among which about 3.54 million died of hyperglycemia (1). The global prevalence of diabetes is estimated at 9.3% (463 million) in 2017 and is predicted to increase to 10.2% (578.8 million) by 2030 (2). Therefore, hyperglycemia is a notably high risk chronic disease (3, 4). Hyperglycemia is preventable through healthy eating, maintaining a normal weight, and physical activity. An intervention study has shown that nutritional supplementation with folic acid, vitamin B_12_, vitamin B_6_, choline, and protein altered the cardiometabolic risk profiles of older adults (5). Therefore, this has aroused considerable interest in identifying the association between one-carbon metabolism nutrients and hyperglycemia.

One-carbon metabolites are a series of interconnected metabolic pathways, including the folate and methionine cycles, which provide methyl groups for the synthesis of DNA (6, 7). DNA methylation plays a key role in cardiovascular disease (CVD) and diabetes. The function of one-carbon metabolites and the availability of adequate methyl groups depend on essential nutrients, such as folate, methionine, choline, betaine, vitamin B_6_, and vitamin B_12_ (8, 9). Many studies have explored the effect of one-carbon metabolism nutrients on diabetes and CVDs (10, 11). However, most studies have drawn inconsistent conclusions whether hyperglycemia, a risk factor for diabetes and CVDs, is affected by one-carbon metabolism nutrients (12–16). Moreover, evidence from choline and betaine is limited. Studies reported hyperglycemia was associated with choline and betaine in Norway (17) and Newfoundland population (18). However, these associations have not been established in Canadian women (19) and American (20). These studies suggested that the results were inconsistent across countries because of dietary habits. Therefore, it needs to be clarified the association between one-carbon metabolism nutrients and hyperglycemia among Chinese. However, no studies have explored the association of habitual dietary intake of choline and betaine and with risk of hyperglycemia in China.

Previous studies observed a positive effect of fluoride on the incidence and prevalence of diabetes in the USA and Canada (21, 22). It was reported that a higher intake of one-carbon metabolism nutrients was associated with a lower occurrence of fluorosis (23). The interaction between one-carbon metabolism nutrients and fluorosis related to glucose levels needs to be clarified.

The present study aims to comprehensively investigate the associations between dietary intake of six one-carbon metabolism nutrients (betaine, choline, methionine, folate, vitamin B_6_ and vitamin B_12_) and hyperglycemia among a Chinese population in areas known for coal-burning fluorosis. The study also explores whether the associations between one-carbon metabolism nutrient intake and hyperglycemia are modified by the presence or absence of fluorosis.

## Methods

### Study population

We used a cross-sectional study design conducted in Zhijin County, Guizhou province, China. A two-stage cluster sampling method was used in this study. The three towns of Chadian, Chengguang, and Puweng were selected by simple random sampling approach from 10 towns in Zhijin County. From each of these towns, we selected four villages at random. The 12 villages were Dazai, Ganhe, Gaofeng, Guihua, Guohua, Hehua, Hualuo, Jiangyan, Shangzai, Yutang, Xianfeng, and Moda. Participants were recruited through village doctors and the Center for Disease Control and Prevention from each selected village. Participants diagnosed with either dental fluorosis or skeletal fluorosis were identified as fluorosis patients. The diagnoses of dental fluorosis (WS/T208-2011, China) and skeletal fluorosis (GB 16396–1996) were performed by a specialist from the CDC. A detailed description of the method is described in our previous studies (24, 25). We used the following participant inclusion criteria: (1) participants must have lived in Zhijin County for at least 10 years; and (2) be between 18 and 75 years old. We used the following exclusion criteria: (1) Participants had a prior history of cancer, coronary heart disease, stroke, gout, or kidney disease; (2) Participants' whose eating habits had changed significantly over the past 5 years; (3) Participants who currently had a chronic medical condition that may affect their eating habits, such as cancer, gout or hypertension; (4) Participants with incomplete questionnaire information. The final analyses involved 901 subjects. We obtained post-fasting blood data from all the 901 participants who attended the interview. All of the subjects in this study were provided written informed consent before the interview. This study protocol was approved by the Ethics Committee of Zunyi Medical University.

### Data collection pertaining to diet and lifestyle

The data that related to sociodemographic characteristics (age, sex, marital status, education level, average yearly household income, ethnicity, smoking status, and alcohol consumption) and disease history (cancer, endemic fluorosis, coronary heart disease, stroke, gout, and kidney disease) were collected by questionnaires. Smokers were defined as those who smoke cigarettes ≥1/day for at least 1 year. Alcohol drinkers were defined as those who drink ≥50 g alcohol/day for at least 1 year.

Dietary nutrient intake information was collected through a valid and reliable food-frequency questionnaire (FFQ). The participants gave frequency of consumption information (never, daily, weekly, monthly, yearly) for each food item over the previous year. The 75-item FFQ involved seven categories including fruits, animal products, cereals, beans, vegetables, algae and nuts, beverage drinks and soups. Pictures of food portion sizes were offered to help participants to estimate the quantity of food consumption. Then portion size of each food was converted to grams. Daily dietary intakes of one-carbon metabolism nutrients were estimated by multiplying the consumption frequency and portion size of each food by the nutrient content based on the Chinese Food Composition Database. We used the Chinese food composition database to calculate the estimated intake of vitamin B_6_, B_12_, folate, and methionine (26). In addition, the USDA choline content database was used to calculate the estimated intake of betaine, choline, and choline subclasses, including free choline, glycerol phosphorylcholine, phosphocholine, phosphatidylcholine, and sphingomyelin (27). Total choline intake was calculated as the sum of choline intake from glycerophosphocholine, phosphocholine, phosphatidylcholine, sphingomyelin and free choline. Previous studies have shown high correlations between USDA and Chinese database estimates for common nutrient intakes. For example, *r* > 0.90 for B vitamins and methionine indicates the validity of using the USDA database in the Chinese population to evaluate the intake of choline and betaine (28).

### Measurement and detection

Waist Circumference (WC), weight, and body height were measured following a standardized protocol, and body mass index (BMI) was calculated as weight divided by height^2^ (kg/m^2^). Weight was measured to the nearest 0.1 kg, and height and WC were measured to the nearest 0.1 cm. Venous blood samples were collected from 7 to 9 am following an overnight fast of at least 8 h. A drop of venous blood was used for bedside measurement of blood glucose, using the Accu-Chek Active glucometer (Roche Diagnostics GmbH, Germany). According to the World Health Organization (WHO) (29), patients with fasting glucose ≥6.1 mmol/L were diagnosed hyperglycemia.

### Statistical analysis

All statistical analyses were performed using SPSS software (version 17.0). All *P*-values were two-sided, and the level of significance was set at 0.05. Continuous variables were reported as means and standard deviations or median values, while categorical variables were expressed as numbers and percentages. Analyses of two independent sample *t*-tests were used to analyze the normally distributed mean differences between the participants' characteristics. The *Mann-Whitney* test was used to compare the median consumption levels, which did not have a normal distribution. Chi square (χ2) tests were used to compare the among dietary frequency categories between hyperglycemic and normal blood glucose level participants. A logistic regression model was applied to estimate the association between dietary intake of one-carbon metabolism nutrients and hyperglycemia. Odds Ratios (ORs) with 95% confidence intervals (95% CIs) were reported for each quartile of one-carbon metabolism nutrient intake. The first quartile was considered as a reference. The multivariable models were adjusted for age, BMI, sex, marital status, family income, education level, smoking status, alcohol consumption, total energy intake, and dietary fiber intake. Moreover, all six nutrients were placed in a single model, with adjustment of the risk factors listed above. Because one-carbon metabolism–related nutrients are thought to influence disease risk by donating methyl groups for methylation reactions (30, 31), we also calculated a “methyl-donor index” as a composite measure of dietary methyl intake by standardizing the nutrient intake levels on the log-scale ([nutrient value – mean] / standard deviation) then summed across all six nutrients, as described previously (32). The methyl-donor index also was adjusted for total calorie intake using the residual method then categorized into quartile. Trend tests were performed by using the sequential values of the quartiles of dietary one-carbon metabolism nutrients as a continuous variable. Moreover, we evaluated if associations were modified by the presence or absence of fluorosis. The multivariable models were adjusted for age, BMI, and gender. Trend tests were conducted by entering the ordinal values of the quartile of dietary one-carbon metabolism nutrients as continuous variables in the models.

## Results

In this cross-sectional study, we collected blood glucose level data from 901 subjects including 417 males (46.28%) and 484 females (53.27%). According to blood glucose levels, they were divided into a hyperglycemic group (*n* = 184, 20.42%) and a normal group (*n* = 717, 79.58%). The general information and measurement indicators are shown in Table 1. The average age of the participant was 49 years old, and the average age of the hyperglycemic group (52.99 ± 12.76) was higher than the normal glucose level group (48.59 ± 13.01). There was a significant difference in age, BMI, and waist circumference between the two groups (*P* < 0.001–*P* = 0.005), although the differences in gender, education level, ethnicity, per capita annual income, marital status, smoking, alcohol consumption, height, weight and fluorosis between the two groups were not observed (*P* > 0.05).

**Table 1:** The general information of participants.

**Characteristics**	**Total** **population** **NO.(%)/Median**	**Hyperglycemia** **(*n* = 184)**	**Normal** **blood glucose** **(*n* = 717)**	***t/X^2^/Z***	***P-*** **value**
^**^Age, year	49.49	52.99 ± 12.76	48.59 ± 13.01	−4.109	<0.001
Gender, (%)				2.147	0.143
Men	417 (46.28)	94 (51.10)	325 (45.00)		
Women	484 (53.27)	90 (48.90)	394 (55.00)		
Marital status, (%)				4.355	0.113
Married or cohabiting	756 (84.09)	152 (82.60)	604 (84.20)		
Divorced or separated	96 (10.67)	26 (14.10)	70 (9.70)		
Unmarried	47 (5.22)	6 (3.30)	41 (5.70)		
Education, (%)				−1.539	0.124
Illiteracy	425 (47.27)	94 (51.10)	331 (46.20)		
Primary school	300 (33.37)	63 (34.20)	237 (33.10)		
Secondary school	131 (14.57)	19 (19.30)	112 (15.60)		
High school or above	43 (4.78)	8 (4.30)	35 (4.80)		
Ethnicity, (%)				0.733	0.693
Han	510 (56.70)	101 (54.90)	409 (57.20)		
Buyei	80 (8.90)	15 (8.20)	65 (9.10)		
Other ethnicity	309 (34.40)	68 (37.00)	241 (33.70)		
Income, (yuan/capita/year), (%)				−0.175	0.861
≤1,000	177 (19.68)	41 (22.30)	136 (19.00)		
1,001–2,000	257 (28.58)	48 (26.00)	209 (29.20)		
2,001–4,000	242 (26.91)	47 (25.50)	195 (27.20)		
4,001–6,000	116 (12.90)	25 (13.60)	91 (12.70)		
>6,000	107 (11.90)	23 (12.50)	84 (11.70)		
Smoker^a^, (%)	365 (40.60)	82 (44.60)	283 (39.60)	1.508	0.219
Alcohol drinker^b^, (%)	278 (30.82)	53 (28.80)	225 (31.40)	0.502	0.479
^**^Height, cm	155.72	155.35 ± 8.26	155.81 ± 8.07	0.696	0.486
^*^Weight, kg	55.00	56.05 (48.30, 65.37)	54.50 (49.50, 61.10)	−1.709	0.087
^*^Body mass index, kg/m^2^	22.53	23.66 (20.57,27.16)	22.46 (20.26,25.10)	−2.837	0.005
^*^Waist circumference, cm	77.00	79.75 (72.00, 88.75)	77.00 (70.00, 84.00)	−3.197	0.001
Fluorosis	657 (72.91)	131 (71.20)	526 (73.40)	0.348	0.555

** Continuous normal data were expressed as Mean ± SD,

* Skewness data were represented by Median (25th, 75th).

^*a*^Smokers were defined as smoking cigarettes ≥1/day on average for at least 1 year.

^*b*^Alcohol drinkers were defined as having drunk ≥50 g/day for one consecutive year or more. SD, Standard Deviation.

Intake of folate, vitamin B_6_, total choline and methyl-donor index in the hyperglycemia group was lower than that in the normal blood glucose level group (*P* = 0.035, *P* = 0.011, *P* = 0.006, *P* = 0.002, respectively). As for the choline subclass, the intake of free choline, glycerol phosphorylcholine, phosphatidylcholine, and phosphocholine had significant differences between the two groups (all *P* < 0.05). Dietary choline intake of the hyperglycemic group was lower than the normal glucose level group. However, no differences in energy intake, vitamin B_12_, methionine, betaine and sphingomyelin intake between the two groups were observed (*P* = 0.074–0.384). The overall intake of one-carbon metabolism nutrients was generally lower in the hyperglycemic group (Table 2).

**Table 2:** Comparison of energy, one-carbon metabolism nutrients intake between hyperglycemia and normal blood.

**Items**	**Hyperglycemia (*n* = 184)**	**Normal blood glucose (*n* = 717)**	***Z/t***	***P-*** **value**
	**Median (25th, 75th)**	**Median (25th, 75th)**		
^*^Energy (kcal/d)	2,718.20 (2,107.35, 3,446.64)	2,521.18 (1,966.43, 3,355.63)	−1.786	0.074
^*^Folate (ug/d)	403.39 (316.08, 521.25)	439.50 (336.62, 561.97)	−2.104	0.035
^*^Vitamin B_6_ (mg/d)	1.44 (1.07, 1.86)	1.57 (1.19, 1.99)	−2.534	0.011
^*^Vitamin B_12_ (ug/d)	1.98 (0.90, 3.04)	2.00 (1.11, 3.18)	−1.145	0.252
^*^Methionine (mg/d)	2,040.94 (1,495.73, 2,586.20)	2,088.86 (1631.59, 2596.95)	−1.080	0.280
^*^Betaine (mg/d)	92.21 (56.97, 148.1)	96.98 (59.34, 167.19)	−0.871	0.384
^*^Total choline (mg/d)	168.49 (118.91, 253.48)	194.03 (134.49, 285.12)	−2.759	0.006
^*^Free choline (mg/d)	43.53 (32.25, 61.95)	50.62 (37.32, 72.62)	−3.795	<0.001
^*^glycerol ^*^phosphorylcholine (mg/d)	16.69 (12.50, 22.71)	19.60 (15.27, 26.28)	−4.275	<0.001
^*^Phosphocholine (mg/d)	7.66 (5.02, 11.41)	9.54 (6.33, 14.06)	−3.964	<0.001
^*^Phosphatidylcholine (mg/d)	91.80 (54.58, 153.29)	103.99 (63.18, 162.28)	−2.010	0.044
^*^Sphingomyelin (mg/d)	4.09 (1.93, 7.51)	4.78 (2.46, 8.27)	−1.718	0.086
^**^methyl-donor index^a^	−1.70 ± 9.39	0.55 ± 8.56	3.118	0.002

The intakes of one-carbon metabolism nutrition and choline subclass were adjusted by the residual method. P25 and P75 represent the 25th and 75th percentiles respectively. SD, Standard Deviation. d, day.

* Mann-Whitney test was used to compare median intake levels in the hyperglycemic and the normal group.

** Two independent sample t-tests were used to analyze mean intake levels in the hyperglycemic and the normal group. Total choline intake is the sum of phosphocholine, free choline, glycerol phosphorylcholine, phosphatidylcholine, and sphingomyelin.

a Sum over the six nutrients of z scores of log of nutrient intake.

The ORs (95% CI) for the occurrence of hyperglycemia according to quartiles of one-carbon metabolism nutrient intake and methyl-donor index are presented in Table 3. After adjusting for age, sex, marital status, education level, average yearly household income, ethnicity, smoking status, and alcohol consumption, we found a greater intake of dietary vitamin B_6_ and total choline were inversely associated with the occurrence of hyperglycemia. The adjusted OR (95% CI) in the highest quartile compared with the lowest was 0.599 (0.365, 0.984) for total choline and 0.579 (0.349, 0.961) for vitamin B_6_. The inverse associations remained after further adjustments for total energy and dietary fiber. There also was a suggestion that methyl-donor index was inversely associated with the occurrence of hyperglycemia, with adjustment for potential confounding factors (*P* = *0.011*). However, there were no significant associations between the intake of folate, vitamin B_12_, methionine, betaine and hyperglycemia after adjusting for non-dietary and dietary factors.

**Table 3:** Odds Ratios (*ORs*) and 95% confidence intervals (95%*CIs*) of hyperglycemia according to quartiles of one-carbon metabolism intake.

**Items**	**Quartiles of one-carbon metabolism nutrients intake**				***P-*** **value**
	**Q1**	**Q2**	**Q3**	**Q4**	
Folate (ug/d)	268.23	381.82	486.97	669.68	
OR (95% CI)	1	0.927 (0.597, 1.440)	0.763 (0.485, 1.199)	0.659 (0.414, 1.050)	0.056
OR (95% CI)^1^	1	0.991 (0.624, 1.575)	0.790 (0.491, 1.272)	0.707 (0.428, 1.168)	0.118
OR (95% CI)^2^	1	0.988 (0.621, 1.572)	0.777 (0.482, 1.253)	0.710 (0.429, 1.174)	0.121
Vitamin B_6_ (mg/d)	0.95	1.36	1.76	2.35	
OR (95% CI)	1	0.861 (0.555, 1.335)	0.790 (0.507, 1.232)	0.550 (0.342, 0.885)^*^	0.012
OR (95% CI)^1^	1	0.852 (0.538, 1.349)	0.774 (0.484, 1.240)	0.579 (0.349, 0.961)^*^	0.037
OR (95% CI)^2^	1	0.841 (0.531, 1.333)	0.780 (0.487, 1.250)	0.575 (0.346, 0.956)^*^	0.038
Vitamin B_12_ (ug/d)	0.66	1.54	2.52	4.16	
OR (95%CI)	1	0.628 (0.395, 0.998)^*^	0.834 (0.537, 1.295)	0.730 (0.465, 1.146)	0.507
OR (95%CI)^1^	1	0.653 (0.403, 1.057)	0.873 (0.547, 1.393)	0.774 (0.478, 1.254)	0.681
OR (95%CI)^2^	1	0.653 (0.402, 1.059)	0.877 (0.549, 1.400)	0.777 (0.479, 1.261)	0.695
Methionine (mg/d)	1,297.23	1,837.71	2„309.96	3,042.96	
OR (95%CI)	1	0.723 (0.458, 1.143)	0.785 (0.500, 1.231)	0.834 (0.533, 1.304)	0.294
OR (95%CI)^1^	1	0.775 (0.481, 1.251)	0.847 (0.526, 1.364)	0.917 (0.562, 1.497)	0.421
OR (95%CI)^2^	1	0.783 (0.485, 1.264)	0.847 (0.525, 1.366)	0.942 (0.576, 1.540)	0.467
Betaine (mg/d)	29.03	76.53	122.34	262.66	
OR (95%CI)	1	1.054 (0.673, 1.649)	0.968 (0.616, 1.523)	0.773 (0.483, 1.237)	0.189
OR (95%CI)^1^	1	1.057 (0.662, 1.688)	0.931 (0.576, 1.507)	0.790 (0.480, 1.302)	0.223
OR (95%CI)^2^	1	1.078 (0.674, 1.724)	0.944 (0.583, 1.530)	0.810 (0.491, 1.337)	0.220
Total choline (mg/d)	100.21	159.50	225.35	376.13	
OR (95%CI)	1	0.720 (0.462, 1.121)	0.696 (0.446, 1.086)	0.567 (0.457, 0.899)^*^	0.015
OR (95%CI)^1^	1	0.718 (0.452, 1.139)	0.693 (0.434, 1.105)	0.599 (0.365, 0.984)^*^	0.032
OR(95%CI)^2^	1	0.723 (0.455, 1.148)	0.695 (0.435, 1.109)	0.601 (0.365, 0.988)^*^	0.032
Methyl-donor index^a^	−9.7251	−2.2502	3.2221	9.8571	
OR (95%CI)	1	0.818 (0.530, 1.233)	0.508 (0.311, 0.832)**	0.666 (0.406,1.094)	0.002
OR (95%CI)^1^	1	0.783 (0.498, 1.352)	0.659 (0.410, 1.059)	0.809 (0.497, 1.317)	0.010
OR(95%CI)^2^	1	0.779 (0.495, 1.227)	0.505 (0.308, 0.827) **	0.671 (0.407, 1.104)	0.011

Q1 was used as the reference, and OR (95%) was unadjusted.OR (95%);

1 adjusted for age, gender, BMI, marital status, family income, smoking status, and alcohol consumption. OR (95%);

2 further adjusted for total energy intake and dietary fiber.

* *P* < 0.05.

a Sum over the six nutrients of z-scores of log of nutrient intake.

The OR (95% CI) for the occurrence of hyperglycemia according to quartiles of five choline intakes subclasses is presented in Table 4. In the unadjusted model, compared with the lowest quartile, the ORs (95% CIs) of hyperglycemia for the highest quartile intakes of free choline, glycerol phosphatidylcholine, and phosphocholine were 0.414 (0.259, 0.661), 0.490 (0.306, 0.783), and 0.420 (0.260, 0.678), respectively. The observed inverse associations remained after further adjustment for potential confounding factors. In contrast, no statistical association between dietary intake of phosphatidylcholine and sphingomyelin and hyperglycemia was observed (*P* > 0.05).

**Table 4:** Odds Ratios (*ORs*) and 95% confidence intervals (95%*CIs*) of hyperglycemic according to quartiles of five choline-containing compounds intake.

**Items**	**Quartiles of five choline-containing compounds intake**				***P-*** **value**
	**Q1**	**Q2**	**Q3**	**Q4**	
Free choline (mg/d)	29.14	42.57	57.45	91.27	
OR (95%CI)	1	0.602 (0.390, 0.930)^*^	0.518 (0.332, 0.930)^**^	0.414 (0.259, 0.661)^**^	0.001
OR (95%CI)^a^	1	0.625 (0.397, 0.984)^*^	0.474 (0.294, 0.762)^**^	0.419 (0.255, 0.690)^**^	0.002
OR (95%CI)^b^	1	0.635 (0.403, 1.002)	0.477 (0.296, 0.768)^**^	0.425 (0.258, 0.700)^**^	0.002
Glycerophosphoryl choline (mg/d)	11.30	16.79	21.88	31.42	
OR (95%CI)	1	0.955 (0.627, 1.454)	0.454 (0.282, 0.731)^*^	0.490 (0.306, 0.783)^**^	<0.001
OR (95%CI)^a^	1	0.941 (0.605, 1.462)	0.409 (0.247, 0.677)^**^	0.485 (0.294, 0.801)^**^	0.001
OR (95%CI)^b^	1	0.941 (0.604, 1.464)	0.407 (0.245, 0.675)^**^	0.496 (0.300, 0.821)^**^	0.002
Phosphocholine (mg/d)	4.28	7.52	10.87	17.91	
OR (95%CI)	1	0.771 (0.502, 1.182)	0.565 (0.361, 0.886)^*^	0.420 (0.260, 0.678)^**^	<0.001
OR (95%CI)^a^	1	0.721 (0.459, 1.132)	0.506 (0.313, 0.818)^**^	0.443 (0.266, 0.736)^**^	0.001
OR (95%CI)^b^	1	0.725 (0.461, 1.139)	0.508 (0.314, 0.823)^**^	0.447 (0.268, 0.743)^**^	0.001
Phosphatidylcholine (mg/d)	44.41	82.11	124.60	223.56	
OR (95%CI)	1	0.792 (0.506, 1.239)	0.766 (0.488, 1.200)	0.706 (0.447, 1.114)	0.116
OR (95%CI)^a^	1	0.792 (0.497, 1.262)	0.750 (0.468, 1.204)	0.773 (0.474, 1.261)	0.177
OR (95%CI)^b^	1	0.794 (0.498, 1.265)	0.752 (0.468, 1.207)	0.765 (0.469, 1.253)	0.175
Sphingomyelin (mg/d)	1.28	3.53	6.37	11.83	
OR (95%CI)	1	0.880 (0.565, 1.371)	0.763 (0.485, 1.199)	0.702 (0.443, 1.112)	0.470
OR (95%CI)^a^	1	0.986 (0.619, 1.572)	0.814 (0.506, 1.308)	0.767 (0.470, 1.253)	0.594
OR (95%CI)^b^	1	0.989 (0.620, 1.577)	0.818 (0.508, 1.317)	0.775 (0.474, 1.267)	0.617

Q1 was used as the reference, and OR (95%) was unadjusted.OR (95%);

a Adjusted for age, gender, BMI, marital status, family income, smoking status, and alcohol consumption. OR (95%);

b ^b^Further adjusted for total energy intake and dietary fiber.

* *P* < 0.05,

** *P* < 0.01.

We further assessed the associations between one-carbon metabolism nutrients and hyperglycemia across subgroups stratified by the presence or absence of fluorosis, shown in Tables 5, 6. We observed that the nutrients folate, vitamin B_6_, total choline, free choline, glycerol phosphorylcholine, and phosphocholine had a protective effect against the occurrence of hyperglycemia in the non-fluorosis subgroup. There were inverse associations of the above nutrients with hyperglycemia in the non-fluorosis subgroup (*P* < 0.05), but not in the fluorosis subgroup (*P* > 0.05). There were significant interactive effects for the above nutrient intakes with fluorosis (*P-interaction* = 0.010 *P*=0.048). There was no significant interactive effect for the other nutrients betaine, methionine, vitamin B_12_, phosphatidylcholine, sphingomyelin, and methyl-donor index with fluorosis (*P-interaction* = 0.077 to 0.527).

**Table 5:** Odds Ratios (OR) and 95% confidence intervals (95%CIs) of hyperglycemia according to quartiles of one-carbon metabolism nutrients by fluorosis.

	**Intake of one-carbon metabolism nutrients**					
	**Q1**	**Q2**	**Q3**	**Q4**	***P^a^***	***P^b^***
Folate (ug/d)						0.041
Fluorosis	1	0.949 (0.561, 1.606)	0.833 (0.478, 1.451)	0.859 (0.486, 1.520)	0.747	
Non-fluorosis	1	1.073 (0.414, 2.777)	0.609 (0.241, 1.540)	0.367 (0.137, 0.984)^*^	0.012	
Betaine (mg/d)						0.114
Fluorosis	1	1.027 (0.597, 1.764)	1.007 (0.578, 1.754)	1.033 (0.589, 1.813)	0.973	
Non-fluorosis	1	1.126 (0.461, 2.752)	0.957 (0.396, 2.313)	0.398 (0.151, 1.049)	0.085	
Methionine (mg/d)						0.425
Fluorosis	1	0.696 (0.405, 1.195)	0.797 (0.463, 1.372)	0.894 (0.505, 1.582)	0.741	
Non-fluorosis	1	1.137 (0.425, 3.044)	0.892 (0.351, 2.269)	0.882 (0.359, 2.165)	0.281	
Vitamin B_6_ (mg/d)						0.039
Fluorosis	1	0.908 (0.542, 1.519)	0.732 (0.419, 1.279)	0.760 (0.430, 1.342)	0.461	
Non-fluorosis	1	0.727 (0.279, 1.892)	0.772 (0.317, 1.880)	0.266 (0.096, 0.733)^*^	0.005	
Vitamin B_12(_ug/d)						0.224
Fluorosis	1	0.724 (0.420, 1.250)	0.997 (0.586, 1.696)	0.689 (0.389, 1.220)	0.303	
Non-fluorosis	1	0.439 (0.165, 1.171)	0.572 (0.232, 1.408)	0.922 (0.395, 2.150)	0.396	
Total choline (mg/d)						0.048
Fluorosis	1	0.811 (0.479, 1.373)	0.639 (0.370, 1.105)	0.820 (0.471, 1.429)	0.376	
Non-fluorosis	1	0.534 (0.215, 1.328)	0.674 (0.274, 1.656)	0.236 (0.087, 0.639) ^*^	0.007	
Methyl-donor index ^c^						0.077
Fluorosis	1	0.871 (0.522, 1.456)	0.474 (0.261, 0.859)^*^	0.904 (0.522, 1.567)	0.983	
Non-fluorosis	1	0.603 (0.236, 1.544)	0.436 (0.176, 1.079)	0.241 (0.089, 0.655)^**^	0.994	

OR (95%), adjusted for age, gender and BMI.

a P-value for linear trend;

b P-value for interaction;

c Methyl-donor index: Sum over the six nutrients of z scores of log of nutrient intake.

* *P* < 0.05,

** *P* < 0.01.

**Table 6:** Odds Ratios (OR) and 95% confidence intervals (95%CIs) of hyperglycemia according to quartiles of five choline-containing compounds by fluorosis.

	**Five choline-containing compounds intakes**				***P*_1_ *^a^***	***P*_2_ *^b^***
**Items (mg/d)**	**Q1**	**Q2**	**Q3**	**Q4**		
Free choline						0.010
Fluorosis	1	0.613 (0.361, 1.041)	0.581 (0.339, 0.997)^*^	0.663 (0.382, 1.152)	0.201	
Non-fluorosis	1	0.627 (0.255, 1.542)	0.283 (0.111, 0.725)^**^	0.115 (0.040, 0.328)^**^	<0.001	
glycerol phosphorylcholine						0.047
Fluorosis	1	0.942 (0.567, 1.565)	0.438 (0.244, 0.787)^**^	0.617 (0.353, 1.081)	0.072	
Non-fluorosis	1	0.902 (0.382, 2.127)	0.369 (0.143, 0.955)^*^	0.262 (0.096, 0.716)^**^	0.002	
Phosphocholine						0.038
Fluorosis	1	0.761 (0.458, 1.264)	0.585 (0.336, 1.091)	0.630 (0.359, 1.103)	0.114	
Non-fluorosis	1	0.629 (0.243, 1.625)	0.371 (0.146, 0.945)^*^	0.162 (0.055, 0.473)^**^	0.001	
Phosphatidylcholine						0.147
Fluorosis	1	0.842 (0.493, 1.438)	0.791 (00.461, 1.357)	0.892 (0.510, 1.558)	0.621	
Non-fluorosis	1	0.631 (0.257, 1.549)	0.574 (0.230, 1.430)	0.440 (0.176, 1.100)	0.062	
Sphingomyelin						0.527
Fluorosis	1	1.017 (0.595, 1.736)	0.906 (0.528, 1.555)	0.727 (0.412, 1.284)	0.418	
Non-fluorosis	1	0.929 (0.387, 2.229)	0.636 (0.255, 1.584)	0.796 (0.327, 1.938)	0.782	

OR (95%): adjusted for age, gender and BMI.

a *P*^1^-value for linear trend;

b *P*^2^-value for interaction.

* *P* < 0.05.

** *P* < 0.01.

## Discussion

In the present study, we found the intake of vitamin B_6_, total choline, methyl-donor index, free choline, glycerol phosphorylcholine, and phosphocholine were inversely associated with the occurrence of hyperglycemia in Guizhou province, China. Additionally, we also observed that there were interactions between one carbon metabolic nutrients and fluorosis, such as vitamin B_6_, total choline, free choline, glycerol phosphorylcholine, and phosphocholine.

### Association between choline and betaine intake and hyperglycemia

Hyperglycemia is an important risk factor for diabetes and CVDs. Limited studies evaluated the association of choline and betaine with hyperglycemia and suggested that the results may be inconsistent due to participants from different countries had different dietary habits (17–20). Previous studies reported there was a positive effect of fluoride on the incidence and prevalence of diabetes (21, 22). It is well known that the interaction between nutrition and environment plays an important role in disease. However, whether there is a different relationship between one-carbon metabolism nutrients and hyperglycemia in coal-burning fluorosis areas and how does fluorosis modify the association needs to be evaluated. Therefore, we conducted this study in fluorosis area. To our knowledge we first found protective associations of total choline, free choline, glycerol phosphorylcholine, and phosphocholine with the occurrence of hyperglycemia in Chinese population. Furthermore, these associations were modified by fluorosis. Studies on the association between dietary intake of choline and betaine and hyperglycemia are limited. Some studies explored the relationship of dietary or blood serum choline and betaine with glucose and type 2 diabetes. A cross-sectional study involving 2,394 subjects reported dietary choline intake was negatively associated with fasting blood glucose levels in Newfoundland (18). A cohort study involving 2,332 men (33) found the highest choline and phosphatidylcholine intake was associated with a 25 and a 41% reduction, respectively, in the risk of type 2 diabetes, compared with the lowest quartile in Finland. Moreover, a randomized clinical trial, reported that compared with placebo, betaine tended to reduce fasting glucose levels but had no effect on glycemia and insulin sensitivity (34). It was indicated that betaine may prevent hyperglycemia, even be a potential therapy for the progression of diabetes-induced hyperglycemia. However, choline can be transformed into betaine and phosphatidylcholine through various chemical reactions in the body, which jointly participate in one-carbon metabolism and affect blood glucose levels. More studies are needed to confirm the association between choline, betaine and blood glucose levels.

### Association between vitamin B_6_ and vitamin B_12_ intake and hyperglycemia

Vitamin B_6_ and B_12_ are important co-factors in the one-carbon metabolic pathway. Jin et al. (35) reported that vitamin B_6_ and B_12_ intake was inversely associated with the risk of diabetes in the National Health and Nutrition Examination Survey (NHANES) of 2007–2016. Compared with the lowest quartile, the ORs (95% CIs) of diabetes for the highest quartile intake of vitamin B_6_ was 0.61 (0.42–0.89), the OR (95% CI) of diabetes for the third quartile of dietary vitamin B_12_ was 0.76 (0.60–0.97). A linear inverse relationship was found between vitamin B_12_ and diabetes, and a nonlinear inverse relationship was found between dietary vitamin B_6_ and diabetes. Several small studies explored the association between vitamin B_6_ and diabetes complications and observed B_6_ may play a protective role against various diabetes complications (36, 37). Consistent with these results, we also found that dietary intake of vitamin B_6_ was a protective factor against the occurrence of hyperglycemia. Unlike vitamin B_6_, very few studies have investigated the independent role of vitamin B_12_ in glucose metabolism. China Stroke Primary Prevention Trial longitudinal analyses showed no association between baseline levels of vitamin B_12_ and a new-onset of diabetes or changes in fasting blood glucose levels (38). Zhu et al. (13) observed no association between the intake of vitamin B_12_ and diabetes incidence. Our results on the effect of vitamin B_12_ on hyperglycemia are consistent with these observations. However, a follow-up south Indian study involving 1,500 individuals (12) reported that the levels of vitamin B_12_ decreased with increasing severity of glucose tolerance. Similar results have been reported in another study (39). The inconsistent results could be attributed to the patients countries. Additionally, baseline serums reflect short-term intake levels, whereas FFQ-based surveys on behavior long-term intake levels. More studies combining dietary intake and blood serum levels are needed to investigate the association between vitamin B_6_/B_12_ and blood glucose.

### Association between folate and methionine intake and hyperglycemia

A number of studies have examined the role of folate on glucose and type 2 diabetes, though the results have been inconsistent. In a meta-analysis of 29 randomized controlled trials (RCTs), Lind et al. (14) found that folate supplementation have no overall effect on fasting glucose levels. Recently, Akbari et al. (40) conducted a meta-analysis on RCTs and also observed no significant changes in fasting blood glucose and HbA1c levels among participants with metabolic diseases after folate supplementation, although folate supplementation resulted in decreased plasma insulin levels and insulin resistance. The present study was consistent with the result of RCTs. However, there are two studies that found an inverse association between dietary folate and diabetes risk in Korean (41) and Japanese (42) adults. The Korean study observed that higher dietary folate intake was associated with a lower risk of developing type 2 diabetes for women but not men (41). However, the present study showed no significant association between folate intake and hyperglycemia and no gender difference (Supplementary Tables 1, 2). The reasons for the different results may relate to the adjustment of confounding factors and the use of folate supplements. Additionally, we found that methionine intake was not associated with the occurrence of hyperglycemia. There are few studies methionine and blood glucose. More epidemiological studies are needed to clarify the association between methionine and hyperglycemia.

### Underlining mechanisms

The effect of one-carbon metabolism nutrients on hyperglycemia may be partly related to DNA methylation in epigenetics. DNA methylation during glucose metabolism was implicated in the pathogenesis of type 2 diabetes (43–45). Deficiency or excess of nutrients can affect one-carbon metabolism, which changes the availability of S-adenosyl methionine (SAM) in the methionine cycle and interferes with DNA and histone methylation patterns (46). holine supplementation (1 mM) increased global DNA methylation and DNA methyltransferase expression in both low-glucose (5 mM) and high-glucose (35 mM) conditions. Choline supplementation increased the expression of peroxisomal acyl-coenzyme A oxidase 1 (ACOX1), which mediates fatty acid β-oxidation, especially in high-glucose conditions. High-glucose exposure increased the transcription of the gluconeogenic gene phosphoenolpyruvate carboxykinase (PEPCK), while choline supplementation mitigated increase. Compared to HepG2 cells, the placenta-derived BeWo cells were relatively unresponsive to either high-glucose or high-choline treatment (47). In a word, choline and glucose interacted to affect macronutrient metabolic genes, yet there was no indication that choline may worsen glycemic control in these *in vitro* human cell culture models. Another mechanism may be related to oxidative stress. Oxidative stress leads to impaired glucose uptake in muscle and fat cells and decreases insulin secretion from beta cells (48, 49). Mitochondrial respiration is the major cellular source of reactive oxygen species (ROS), and this production is balanced by through antioxidant systems superoxide dismutase (SOD). In hyperglycemic states, such as prediabetes and diabetes, ROS can accumulate and lead to non-specific oxidative damage to DNA (50). One-carbon metabolism nutrients can directly scavenge reactive oxygen species and can act as an antioxidant *in vivo* (51). In a word, insufficient intake of dietary one-carbon metabolism nutrients may affect the methylation and of ROS, which may prevent the occurrence of hyperglycemia.

### Subgroup analysis

We evaluated the associations whether or not be modified by fluorosis due to this cross-sectional study was conducted in fluorosis area. In the present study, we first observed the interactions between vitamin B_6_, total choline, etc and fluorosis. The result showed that the nutrients folate, vitamin B_6_, total choline, free choline, glycerol phosphorylcholine, and phosphocholine had a protective effect on the occurrence of hyperglycemia in the non-fluorosis subgroup, but not in the fluorosis subgroup. Two studies found that fluoride was significantly and positively associated with increases in both the incidence and prevalence of diabetes (21, 22). Previous animal studies have shown that exposure to high levels of fluoride would decrease insulin mRNA and its secretion from beta-cells, and affect the OGTT (52). Thus, the protective effect hyperglycemia disappeared due to the toxicity of fluorosis. Fluorosis decreased superoxide dismutase (SOD) activity, accompanied by an increase in the generation of superoxide anion and decreased mitochondrial membrane potential in fluoride exposed cells (52). However, one-carbon metabolism nutrients may influence fluorosis by means of their antioxidant properties, as they are well documented antioxidant compounds that reduce the risk of diseases (53, 54). Furthermore, DNA methylation plays an important role in fluorosis (55), one-carbon metabolism nutrients as methyl donors may prevent the change of DNA methylation induced by fluoride. Further studies are warranted to elucidate the competing pathophysiological mechanisms.

### Limitations

The present study has several limitations. First, it did not clearly identify the causal relationship between one-carbon metabolism-related nutrients and hyperglycemia. This was in part because of a cross-sectional study design, even though we minimized the potential reverse causation by excluding participants with other diseases. Second, we could not avoid memory recall bias, although we emphasized unbiased investigative techniques and objective data, discrepancies could exist between what answer was given in the questionnaire and real life behaviors. Thirdly, we cannot exclude the influence of fluorosis, but the subgroup analysis showed that the protective effect was observed in non-fluorosis subgroup against the occurrence of hyperglycemia was consistent with our participant group as a whole. Additionally, our findings generalized to brick-tea or drinking water fluorosis affected areas should be with caution due to this study was conducted in coal-burning fluorosis areas. Fourthly, serum levels of the one-carbon metabolism nutrients were not detected because of traumatic, low-cooperative and expensive, but we investigated dietary intake in a non-invasive and low-cost manner using an FFQ. Finally, we only collected sedentary frequency without detailed physical activity level, so only sedentary frequency was adjusted to control for the effect of activity level. Furthermore, although we adjusted for many factors during statistical analysis, residual confounding factors were still unavoidable.

## Conclusions

In conclusion, higher dietary intakes of vitamin B_6_, total choline, free choline, glycerol phosphorylcholine, and phosphocholine in choline subgroups are associated with a lower incidence of hyperglycemia. This protective effect was modified by the presence or absence of fluorosis. This finding provides a nutritional basis for the prevention of hyperglycemia in patients with fluorosis.

## Data availability statement

The original contributions presented in the study are included in the article/Supplementary material, further inquiries can be directed to the corresponding author/s.

## Ethics statement

The studies involving human participants were reviewed and approved by Zunyi Medical University. The patients/participants provided their written informed consent to participate in this study.

## Author contributions

LD was responsible for analysis and wrote the first draft of the manuscript. QY responsible for data management and revised manuscript. ZS, LL, and ZM assisted in conducting research and data collection. NT carried out the survey and determination. XZ was responsible for organization and survey. JL designed and conducted the study and revised the manuscript. All authors contributed to the article and approved the submitted version.

## Funding

This study was supported by the National Natural Science Foundation of China (grant number 82060595), Guizhou Provincial Natural Science Foundation (QKHJC-ZK[2022]YB598 and QKHJCZK[2022]YB663), and Guizhou Provincial Health Commission Foundation (gzwkj2021-411).

## Conflict of interest

The authors declare that the research was conducted in the absence of any commercial or financial relationships that could be construed as a potential conflict of interest.

## Publisher's note

All claims expressed in this article are solely those of the authors and do not necessarily represent those of their affiliated organizations, or those of the publisher, the editors and the reviewers. Any product that may be evaluated in this article, or claim that may be made by its manufacturer, is not guaranteed or endorsed by the publisher.
