# Supplementary material for: Association between dietary intake of one-carbon metabolism nutrients and hyperglycemia in coal-burning fluorosis areas of Guizhou, China
Source: Front Nutr. 2022 Oct 10;9:1002044. doi: 10.3389/fnut.2022.1002044 (PMC9589113; doi:10.3389/fnut.2022.1002044)
Supplement: Supplementary file 1 [file Data_Sheet_1.docx]

Supplementary Material

Supplementary table 1 Odds Ratios (OR) and 95% confidence intervals (95%CIs) of hyperglycemia according to quartiles of one-carbon metabolism nutrients by gender

|  | | Intake of One-carbon metabolism nutrients | | |  |  |
| --- | --- | --- | --- | --- | --- | --- |
|  | Q1 | Q2 | Q3 | Q4 | *P* _1_*^a^* | *P* _2_ *^b^* |
| Folate (ug/d) |  |  |  |  |  | 0.388 |
| Women | 1 | 1.019 (0.5560, 1.851) | 0.776 (0.402, 1.497) | 0.694 (0.340, 1.417) | 0.133 |  |
| Men | 1 | 0.935 (0.462, 1.891) | 0.787(0.397, 1.561) | 0.682 (0.339, 1.369) | 0.467 |  |
| Betaine (mg/d) |  |  |  |  |  | 0.098 |
| Women | 1 | 1.302 (0.704, 2.410) | 1.150 (0.593, 2.230) | 0.696 (0.338, 1.432) | 0.061 |  |
| Men | 1 | 0.856 (0.427,1.715) | 0.936 (0.482, 1.819) | 0.885 (0.452, 1.735) | 0.915 |  |
| Methionine (mg/d) |  |  |  |  |  | 0.337 |
| Women | 1 | 0.859 (0.459, 1.606) | 1.039 (0.559, 1.931) | 0.859 (0.418, 1.762) | 0.286 |  |
| Men | 1 | 0.654 (0.318, 1.347) | 0.614 (0.301, 1.254) | 0.826 (0.425, 1.606) | 0.901 |  |
| Vitamin B_6_ (mg/d) |  |  |  |  |  | 0.506 |
| Women | 1 | 0.802 (0.441, 1.460) | 0.968 (0.511, 1.833) | 0.478 (0.230, 0.995) ^*^ | 0.082 |  |
| Men | 1 | 0.905 (0.452, 1.811) | 0.686 (0.350, 1.344) | 0.654 (0.325, 1.315) | 0.244 |  |
| Vitamin B_12 (_ug/d) |  |  |  |  |  | 0.700 |
| Women | 1 | 0.660 (0.343, 1.271) | 1.012 (0.514, 1.891) | 0.975 (0.504, 1.887) | 0.623 |  |
| Men | 1 | 0.600 (0.300, 1.201) | 0.716 (0.368, 1.389) | 0.610 (0.313, 1.189) | 0.937 |  |
| Total choline (mg/d) |  |  |  |  |  | 0.863 |
| Women | 1 | 0.953 (0.522, 1.738) | 0.624 (0.319, 1.222) | 0.773 (0.387, 1.544) | 0.159 |  |
| Men | 1 | 0.555 (0.277, 1.113) | 0.713(0.376, 1.349) | 0.467 (0.244, 0.931)^*^ | 0.119 |  |

Note: OR (95%): adjusted for age and BMI. ^a^ *P*-value for linear trend; ^b^P-value for interaction. **P*＜0.05.

Supplementary table 2 Odds Ratios (OR) and 95% Confidence Intervals (95%CIs) of hyperglycemia according to quartiles of five choline-containing compounds by gender

|  | Five choline-containing compounds intakes | | | | *P* _1_*^a^* | *P* _2_ *^b^* |
| --- | --- | --- | --- | --- | --- | --- |
| Items (mg/d) | Q1 | Q2 | Q3 | Q4 |  |  |
| Free choline |  |  |  |  |  | 0.558 |
| Women | 1 | 0.761 (0.413, 1.401) | 0.668 (0.354, 1.261) | 0.379 (0.183, 0.784) ^**^ | 0.016 |  |
| Men | 1 | 0.562 (0.291, 1.085) | 0.409 (0.210, 0.797)^**^ | 0.468 (0.241, 0.910)^*^ | 0.053 |  |
| glycerol phosphorylcholine |  |  |  |  |  | 0.938 |
| Women | 1 | 1.022 (0.567, 1.844) | 0.469 (0.239, 0.917)^*^ | 0.509 (0.248, 1.042) | 0.042 |  |
| Men | 1 | 0.874 (0.461, 1.656) | 0.393 (0.190, 0.813) ^*^ | 0.483 (0.245, 0.951) ^*^ | 0.020 |  |
| Phosphocholine |  |  |  |  |  | 0.245 |
| Women | 1 | 0.700 (0.390, 1.258) | 0.421 (0.212, 0.835) ^*^ | 0.354 (0.171, 0.731) ^**^ | 0.004 |  |
| Men | 1 | 0.842 (0.432, 1.642) | 0.747 (0.390, 1.431) | 0.603 (0.303,1.198) | 0.091 |  |
| Phosphatidylcholine |  |  |  |  |  | 0.959 |
| Women | 1 | 0.992 (0.532, 1.848) | 0.764 (0.398, 1.467) | 0.951 (0.475, 1.906) | 0.464 |  |
| Men | 1 | 0.643 (0.326, 1.266) | 0.730 (0.377, 1.414) | 0.617 (0.322, 1.183) | 0.254 |  |
| Sphingomyelin |  |  |  |  |  | 0.725 |
| Women | 1 | 1.168 (0.637, 2.141) | 0.786 (0.407, 1.520) | 0.826 (0.407, 1.678) | 0.601 |  |
| Men | 1 | 0.842 (0.421, 1.684) | 0.876 (0.452, 1.700) | 0.702 (0.366, 1.346) | 0.763 |  |

Note: OR (95%): adjusted for age and BMI. ^a^ *P*-value for linear trend; ^b^P-value for interaction. **P*＜0.05. ***P*＜0.01
